# Carbamazepine Prevents Hippocampal Neurodegeneration in Mice Lacking the Neuroprotective Protein, Carboxypetidase E

**DOI:** 10.4172/2167-065X.S1-002

**Published:** 2012-10-31

**Authors:** Alicja Woronowicz, Niamh X Cawley, Y Peng Loh

**Affiliations:** Section on Cellular Neurobiology, Program in Developmental Neuroscience, Eunice Kennedy Shriver National Institute on Child Health and Human Development, National Institutes of Health, USA

**Keywords:** Maternal separation, Weaning, Stress, Neurodegeneration, Epilepsy

## Abstract

Carboxypeptidase E (CPE) has recently been described as a neuroprotective protein, and in mice devoid of CPE, a complete loss of the hippocampal CA3 neurons is observed. The pattern of loss is characteristic of that caused by status epilepticus. We therefore set out to determine when this loss occurred, what might induce it and if it could be prevented. We found that the hippocampus was intact in 4 week old CPE knock out (KO) mice that had not undergone weaning. However, weaning of 2 or 3 week old CPE KO mice, which involves maternal separation (emotional stress) and ear tagging and tail snipping for genotyping (physical stress), resulted in degeneration of the CA3 neurons by 3 and 4 weeks of age, respectively, while the wild-type mice were unaffected. Moreover, the physical stress caused a more severe neurodegeneration phenotype than the emotional stress of the maternal separation alone. Daily treatment with carbamazepine, an antiepileptic agent, in 2 week old CPE KO mice for 2 weeks prevented the neurodegeneration, despite the weaning process at 3 weeks. No further neurodegeneration was observed 3 weeks post weaning in carbamazepine treated mice. These results showed that degeneration of the CA3 neurons in the hippocampus, previously observed in 6 week old CPE KO mice, is not due to a developmental defect, but caused by physical and emotional stress during the weaning process. This degeneration was prevented by carbamazepine suggesting that the stress associated with weaning caused epileptic-like events in the CPE KO mice.

## Introduction

Carboxypeptidase E (CPE) was originally characterized as an enzyme that cleaves the C-terminal lysines and arginines from peptide hormone intermediates such as enkephalin [1,2]. Since then, its presence in the endocrine and central nervous systems has been extensively studied and its role in the production of many neuropeptides and peptide hormones, such as α-MSH and insulin is well documented [3]. Indeed, a defect in CPE activity is associated with type 2 diabetes mellitus in humans [4] and in the cathepsin B and L double knockout mice, where early-onset neurodegeneration is observed as a model of neuronal ceroid lipofuscinoses in humans, CPE is increased >10 fold [5], presumably to compensate for the lack of the two enzymes. In addition, in a mouse model for multiple sclerosis, experimental autoimmune encephalomyelitis (EAE), a trait locus for EAE has been mapped to the *Cpe* gene on chromosome 8 [6], while microarray data of inflamed spinal cord of EAE mice showed a concomitant decrease in CPE with an increase in the severity of the disease [7].

In rat brain, CPE levels increase as a response to global and focal ischemic stress [8,9]. In addition, CPE levels increase in the hippocampus under mild chronic restrained stress (Murthy et al., manuscript in preparation) and in the amygdala of rats exposed to the stress of predator odor [10], all suggestive of a protective role of CPE under times of stress. Indeed, over-expression of CPE in hippocampal neurons is neuroprotective against H_2_O_2_ induced oxidative stress induced cell death [11,12]. Hence CPE, a prohormone/proneuropeptide processing enzyme, is up-regulated in neurons in times of stress and appears to function as a neuroprotective molecule.

CPE Knock Out (KO) mice exhibit various endocrine disorders including diabetes, obesity, infertility, and low bone density; they also exhibit neurological and behavioral deficits such as poor learning and memory consolidation [11,13] (for review see [3]). Previously, we reported that 6 week old CPE KO showed a complete loss of hippocampal pyramidal neurons in the CA3 region [11], which could explain these specific neurological deficits. This pattern of neurodegeneration in the hippocampus is characteristic of that observed after an epileptic-like seizure [14]. Studies have shown that stress, which elevates glucocorticoids could render the CA3 region of the hippocampus more susceptible to the onset of epilepsy [15,16]. In this study, we determined at what age and what stress factors might induce degeneration of the CA3 hippocampal neurons in mice lacking CPE. We also investigated whether an antiepileptic agent, carbamazepine, administered to CPE KO mice could prevent the degeneration of these neurons.

## Materials and Methods

### Animals

Colonies of WT and *Cpe* KO mice were housed at the NIH animal facility in groups of 2–5 under a 14:10 h light-dark cycle in a temperature- (22°C) and humidity- (45%) controlled room and provided food and water ad libitum. All experiments were conducted following approved protocols of the Animal Care and Use Committee, NICHD, NIH.

### Weaning

The standard weaning process was performed at 3 weeks of age. During the process, pups were separated from the mother (emotional stress), their ear tagged and the ends of the tails snipped (about 1–2 mm) for genotyping. No anesthesia was used. Different groups of WT or CPE KO mouse pups at 2 or 3 weeks of age were subjected to the standard weaning process, or to the physical stress, or emotional stress only, or not weaned at all. One week after the weaning paradigm, the mice were sacrificed and the hippocampi of the mice were examined. The analysis of hematoxylin and eosin (H&E) stained brain sections were prepared according to methods described previously [11]. Five mice per group for each paradigm were analyzed.

### Treatment of mice with Carbamazepine (CBZ)

One group of 2 week old CPE KO mice (5 pups) was administered 50 mg/kg dose of CBZ orally, once a day, in the form of a 1% solution of Tween 80 suspension in normal saline. Another group (5 pups) was given a 1% solution of Tween 80 in normal saline (vehicle) as the control group. This treatment continued for 2 weeks. At 3 weeks of age the mice were weaned and after 1 more week (at age 4 weeks), the animals were sacrificed and the brains analyzed. Another two groups of mice (5 pups each) were treated and weaned as described above but the animals from these groups were sacrificed at 6 weeks of age.

## Results and Discussion

**Figure 1:**
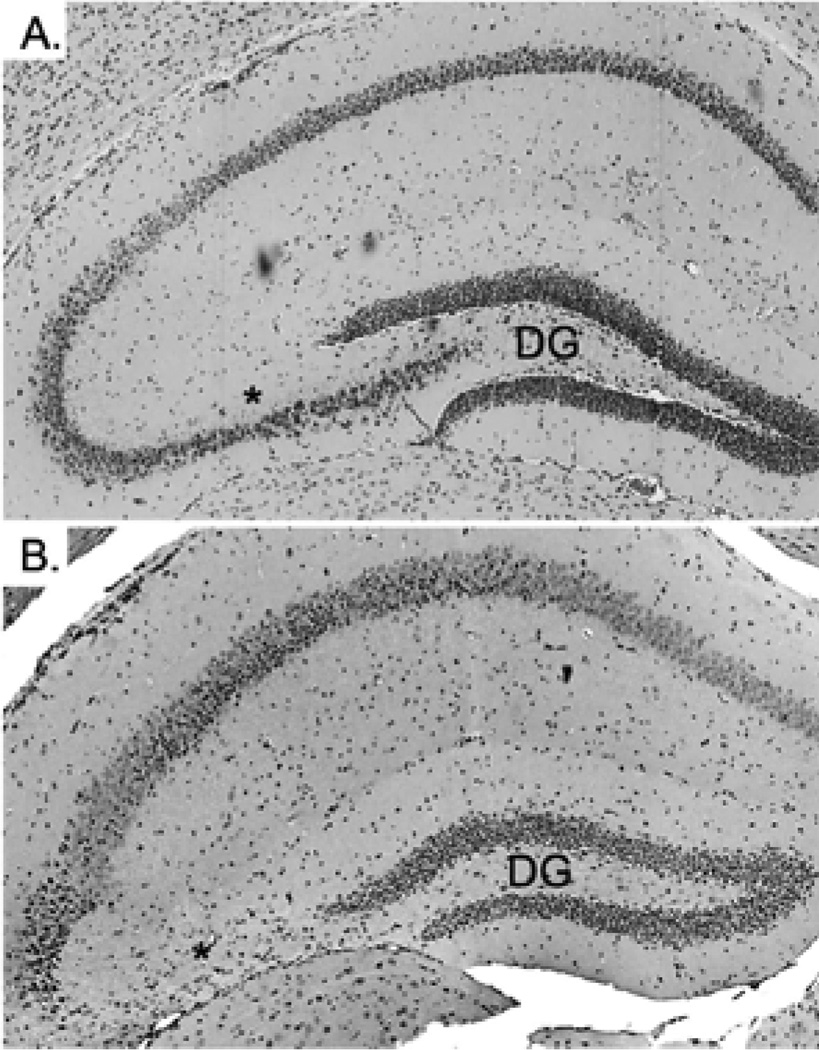
Weaning of CPE KO mice at 2 weeks of age causes degeneration of hippocampal CA3 pyramidal neurons. **A.** WT mice were weaned at 2 weeks of age and analyzed at 3 weeks. The neurons in the CA3 region are intact. **B.** CPE KO mice weaned at 2 weeks of age and analyzed at 3 weeks. Note the lack of pyramidal neurons in the CA3 region. * indicates the CA3 region. Data is representative of 5 animals. DG; Dentate Gyrus.

**Figure 2:**
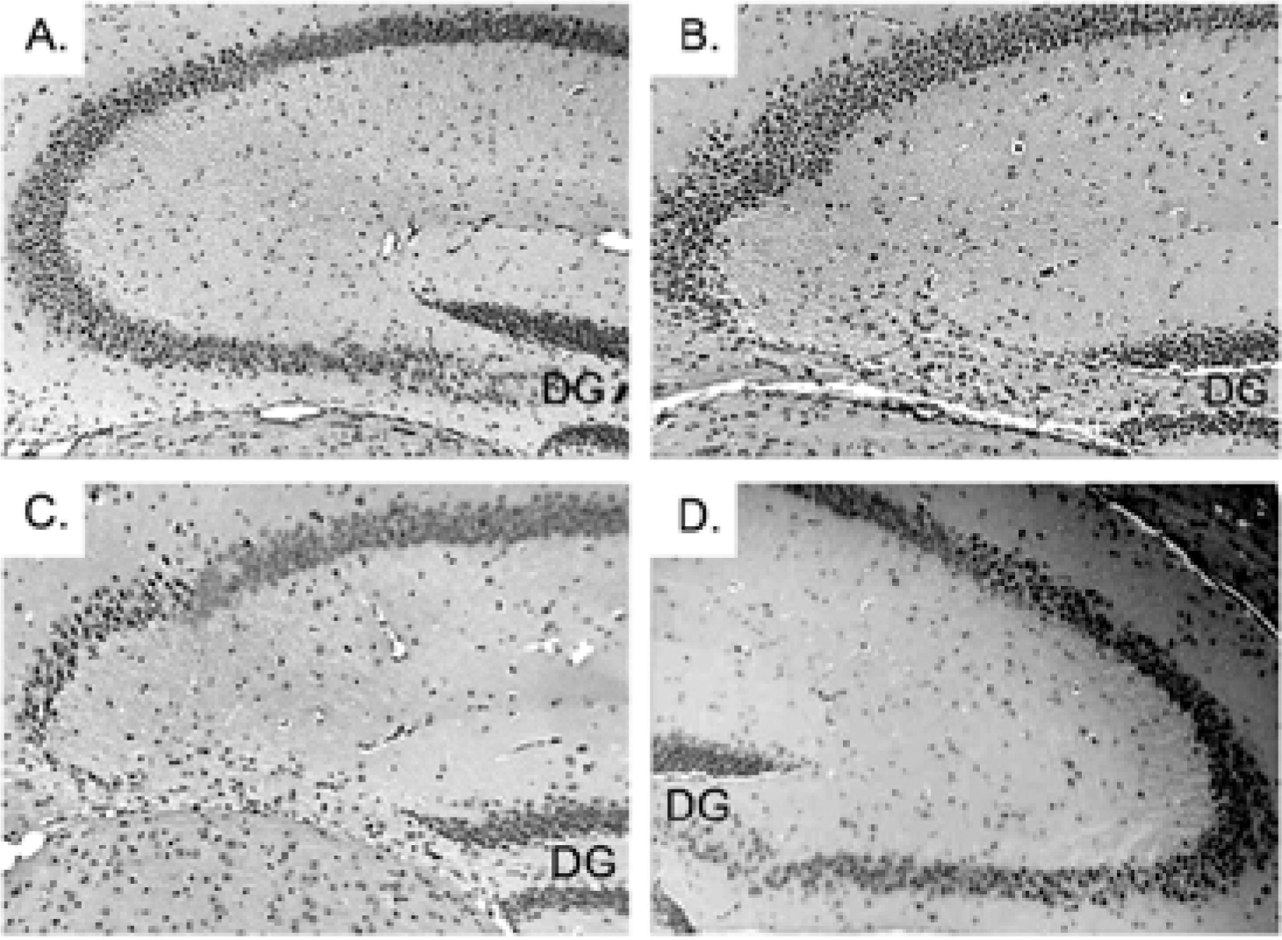
The impact of stress caused by weaning on the survival of pyramidal neurons of the CA3 region of hippocampus in CPE KO mouse pups. **A.** Not weaned (the pyramidal neurons are present). **B.** Physical stress and kept with mother. **C.** Regular weaning with emotional and physical stress (note the degeneration of pyramidal neurons). **D.** Taken from mother (emotional stress) but without physical stress. Note the CA3 neurons are partially degenerated. * indicates the CA3 region. Data is representative of 5 animals. DG; Dentate Gyrus.

The hippocampus of WT and CPE KO mice were examined by hematoxylin and eosin (H&E) staining at 4 weeks of age without weaning the animals. Both WT (data not shown) and CPE KO hippocampi (Figure 2A) were morphologically normal suggesting that the degenerated CA3 neurons observed previously in 6 week old CPE KO animals were not due to a developmental defect of the hippocampus. Rather, we hypothesized that the stress involved with weaning the young animals could induce the neurodegeneration in these KO mice. The weaning process, which routinely occurs at 3 weeks of age as a matter of standard animal husbandry procedures, involves separation of the pups from the mother and transfer into a new cage (maternal separation stress), and ear tagging and tail snipping for genotyping (physical stress). To determine if this weaning stress could cause the degeneration of the CA3 neurons, CPE KO and WT mice were first weaned at 2 weeks of age. Degeneration of the CA3 neurons was observed 1 week later in the CPE KO mice (Figure 1B), whereas similarly treated WT mice were unaffected (Figure 1A). When weaned at 3 weeks, degeneration of the CA3 neurons in the KO mice was observed at 4 weeks of age (Figure 2C) in contrast to KO mice not weaned at 3 weeks of age that showed an intact hippocampus at 4 weeks (Figure 2A).

To determine if either emotional or physical stress alone could induce the neurodegeneration, CPE KO pups were subjected to different weaning paradigms. At 3 weeks of age, the control group of pups was neither separated from the mother, nor underwent the physical stress of ear tagging and tail snipping (Figure 2A). The other three groups went through either only the physical stress, but were kept with the mother (Figure 2B), or only the emotional stress by being removed to a new cage (Figure 2D), or was subjected to both the physical and the emotional stress (Figure 2C). H&E staining of the hippocampi from each group at 4 weeks of age showed that physical stress was a potent factor in causing the degeneration of the pyramidal neurons in the CA3 region in CPE KO mice (Figure 2B). The separation from the mother also resulted in CA3 neuronal degeneration, but to a lesser extent than the physical stress (Figure 2D), perhaps because the emotional stress could be partially alleviated by the pups being transferred to a cage with their siblings. However, CPE KO mice that underwent emotional and physical stress showed complete degeneration of the CA3 neurons (Figure 2C), whereas the mice that did not undergo the weaning process had an intact hippocampus (Figure 2A).

**Figure 3:**
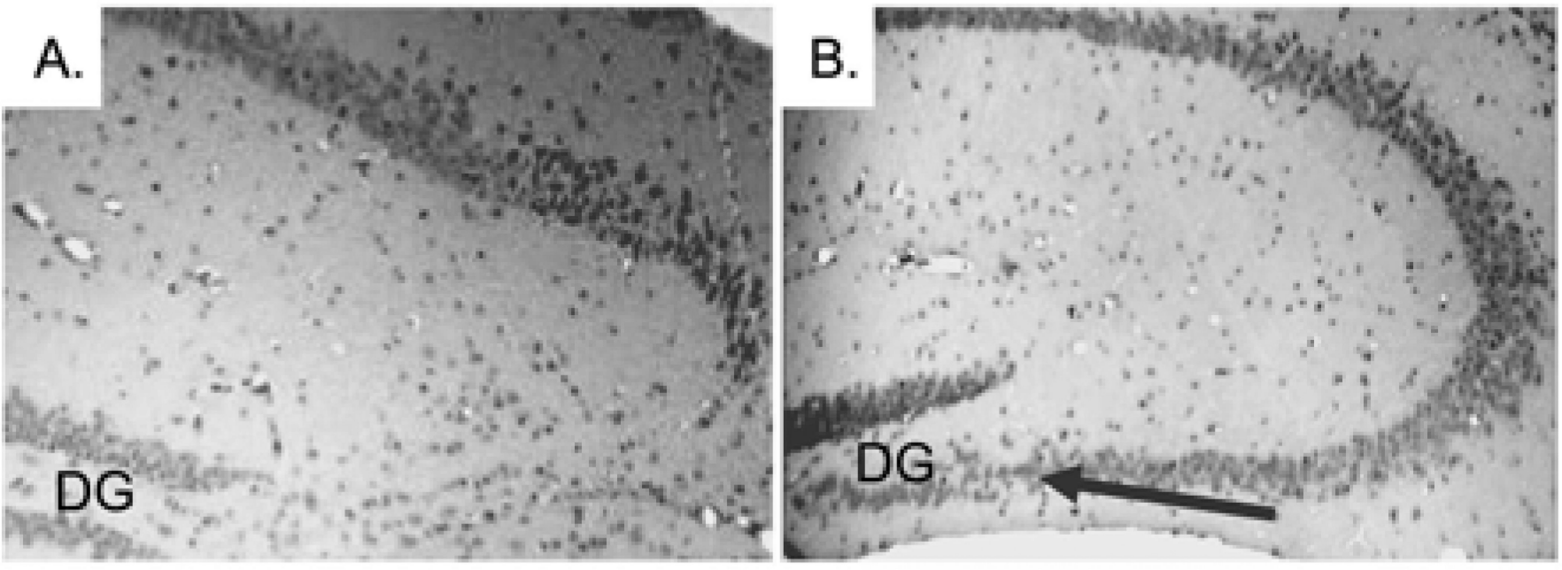
Treatment of 2 week old CPE KO pups daily for 2 weeks with an antiepileptic agent, carbamazepine (CBZ), rescues hippocampal CA3 pyramidal neurons from degeneration with standard weaning stress at 3 weeks. **A.** Normal weaning without CBZ treatment analyzed at 4 weeks. **B.** Normal weaning with CBZ treatment analyzed at 4 weeks. The arrow shows surviving CA3 neurons. Data is representative of 5 animals. DG; Dentate Gyrus.

**Figure 4:**
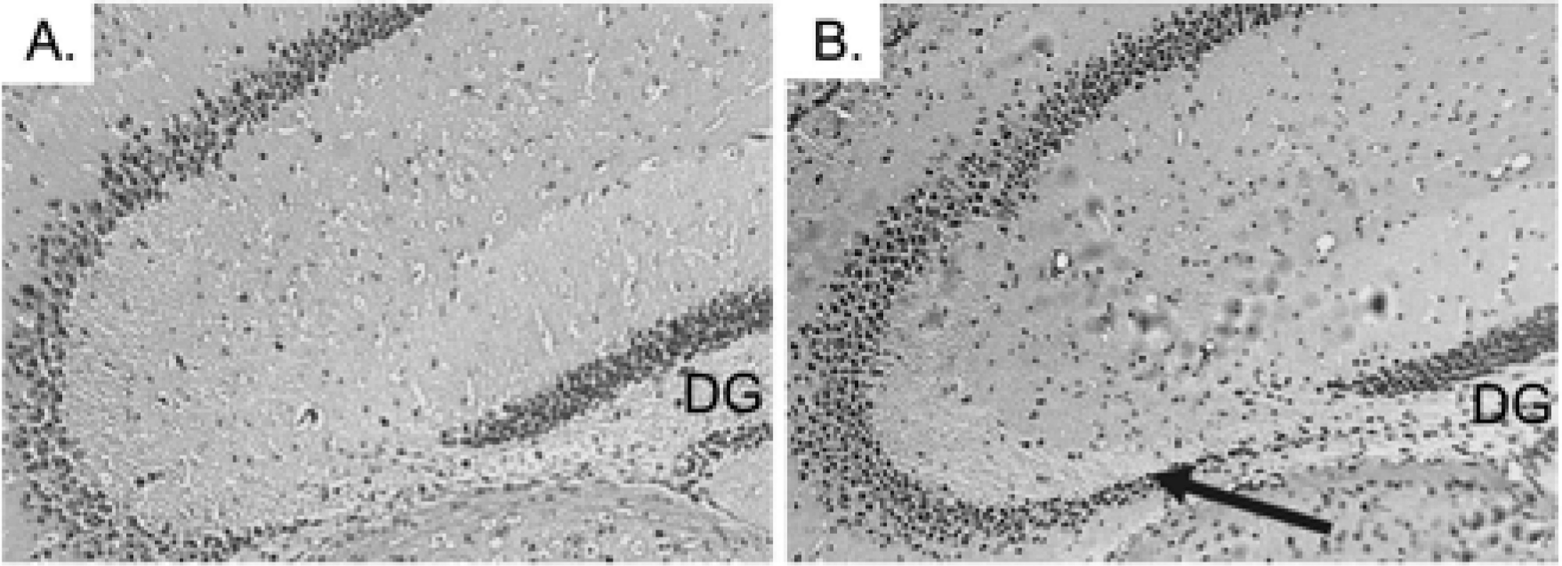
CPE KO pups treated with Carbamazepine (CBZ), as indicated in figure 3 legend, maintain intact hippocampal CA3 pyramidal neurons for 2 weeks after CBZ treatment. **A.** Normal weaning without CBZ treatment and analyzed at 6 weeks of age. **B.** Normal weaning with CBZ treatment for 2 weeks and analyzed at 6 weeks age. The arrow shows surviving CA3 neurons. Data is representative of 5 animals. DG; Dentate Gyrus.

The histopathological abnormalities of the CA3 region in the CPE KO mice after weaning are characteristic of one’s caused by status epilepticus [14]. The similarities suggest that emotional stress due to separation from the mother and physical stress involved with ear tagging and tail snipping might trigger epileptic-like episodes, which contribute to the degeneration of the pyramidal neurons lacking CPE. To test this hypothesis, carbamazepine (CBZ), an antiepileptic agent was given orally to CPE KO mice to determine if it could prevent degeneration of the CA3 pyramidal neurons. CBZ is an anticonvulsant and mood stabilizing drug used primarily in the treatment of epilepsy and bipolar disorders [17]. CBZ stabilizes the inactivated state of sodium channels, which renders the neurons less excitable, and prevents excessive firing [18]. CBZ or vehicle (control) was given orally to CPE KO mice. The treatment started at 2 weeks of age and continued daily for 2 weeks which encompassed the routine weaning at 3 weeks of age followed by analysis at 4 weeks. Analysis of the coronal sections of H&E stained brain tissue revealed that the CA3 region of the CBZ treated CPE KO mice (Figure 3B) was intact after weaning, tail snipping and ear tagging compared to the untreated mice (Figure 3A), demonstrating that CBZ treatment prevented the neuronal loss triggered by weaning. A similarly treated group of CPE KO mice were kept for 2 weeks longer, after the 2 weeks of treatment, and were sacrificed at 6 weeks of age. The results from these mice showed that the protective effect of the CBZ is prolonged, if not permanent (Figure 4B versus Figure 4A untreated control mice).

This study suggests that there may be a critical period in the early life of mouse pups when emotional (maternal separation) and physical stress can trigger epileptic-like episodes of excessive firing of pyramidal neurons. While in WT mice these neurons are protected by CPE, in the KO mice lacking this neuroprotective protein, they degenerate. Treatment of the CPE KO mice with CBZ presumably suppressed the epileptic-like episodes and prevented the neuronal degeneration. Research on association between stress and epileptogenesis in the hippocampus has shown that stress hormones such as glucocorticoids render the entire areas of the limbic system and in particular the CA3 region of the hippocampus, more susceptible to the onset of epilepsy by changing the brain circuits, and altering cell properties and synaptic connections [19,20]. In summary, our studies using the CPE KO mouse model strongly suggests that emotional and physical stress in early life lowers the seizure threshold and exacerbates degeneration of the susceptible neurons in the CA3 region of the hippocampus in the absence of the neuroprotective protein, CPE. These findings further emphasize the importance of CPE as a neuroprotective protein during stress and future studies will be aimed at understanding the mechanism of how CPE carries out this function.
